# A novel rice C2H2-type zinc finger protein, ZFP36, is a key player involved in abscisic acid-induced antioxidant defence and oxidative stress tolerance in rice

**DOI:** 10.1093/jxb/eru313

**Published:** 2014-07-28

**Authors:** Hong Zhang, Yanpei Liu, Feng Wen, Dongmei Yao, Lu Wang, Jin Guo, Lan Ni, Aying Zhang, Mingpu Tan, Mingyi Jiang

**Affiliations:** ^1^College of Life Sciences, Nanjing Agricultural University, Nanjing 210095, People’s Republic of China; ^2^National Key Laboratory of Crop Genetics and Germplasm Enhancement, Nanjing Agricultural University, Nanjing 210095, People’s Republic of China

**Keywords:** Abscisic acid (ABA), antioxidant defence, C2H2-type zinc finger protein, oxidative stress, rice, water stress.

## Abstract

C2H2-type zinc finger proteins (ZFPs) have been shown to play important roles in the responses of plants to oxidative and abiotic stresses, and different members of this family might have different roles during stresses. Here a novel abscisic acid (ABA)- and hydrogen peroxide (H_2_O_2_)-responsive C2H2-type ZFP gene, *ZFP36*, is identified in rice. The analyses of *ZFP36*-overexpressing and silenced transgenic rice plants showed that ZFP36 is involved in ABA-induced up-regulation of the expression and the activities of superoxide dismutase (SOD) and ascorbate peroxidase (APX). Overexpression of *ZFP36* in rice plants was found to elevate the activities of antioxidant enzymes and to enhance the tolerance of rice plants to water stress and oxidative stress. In contrast, an RNA interference (RNAi) mutant of *ZFP36* had lower activities of antioxidant enzymes and was more sensitive to water stress and oxidative stress. ABA-induced H_2_O_2_ production and ABA-activated mitogen-activated protein kinases (MAPKs) were shown to regulate the expression of *ZFP36* in ABA signalling. On the other hand, *ZFP36* also regulated the expression of NADPH oxidase genes, the production of H_2_O_2_, and the expression of *OsMPK* genes in ABA signalling. These results indicate that ZFP36 is required for ABA-induced antioxidant defence, for the tolerance of rice plants to water stress and oxidative stress, and for the regulation of the cross-talk between NADPH oxidase, H_2_O_2_, and MAPK in ABA signalling.

## Summary

In this study it was found that ZFP36 is required for ABA-induced antioxidant defence and for the regulation of the cross-talk between NADPH oxidase, H_2_O_2_, and MAPK in ABA signalling.

## Introduction

Water stress, including drought and salinity, is one of the most important environmental factors that adversely affect plant growth and crop production. Plant adaptation to water stress is dependent on the activation of cascades of molecular networks involved in stress perception, signal transduction, and the expression of specific stress-related genes and metabolites (Huang *et al.*, 2012). The plant hormone abscisic acid (ABA), accumulated in plant cells exposed to water stress, is the central regulator of water stress resistance in plants, and coordinates a complex regulatory network enabling plants to cope with water stress conditions (Cutler *et al.*, 2010; Hubbard *et al.*, 2010; Umezawa *et al.*, 2010; Fujita *et al.*, 2011). One mode of ABA-enhanced water stress tolerance is associated with the induction of antioxidant defence systems, including enzymatic and non-enzymatic constituents, which protect plant cells against oxidative damage. In ABA signal transduction, several signal molecules such as calcium ion (Ca^2+^), reactive oxygen species (ROS), and nitric oxide (NO), and protein kinases such as mitogen-activated protein kinase (MAPK), calcium-dependent protein kinase (CDPK), and calcium/calmodulin-dependent protein kinase (CCaMK) have been shown to play important roles in the regulation of antioxidant defence systems (Jiang and Zhang, 2001, 2002a, *b*, 2003; Zhang *et al.*, 2006, 2007; Neill *et al.*, 2008; Xing *et al.*, 2008; Ye *et al.*, 2011; Ma *et al.*, 2012; Shi *et al.*, 2012, 2014; Ding *et al.*, 2013). However, the mechanisms by which these components regulate antioxidant defence in ABA signalling remain to be determined.

Transcriptional modulation is thought to be one of the most important ways in which plants respond and adapt to stress conditions (Yamaguchi-Shinozaki and Shinozaki, 2006; Nakashima *et al.* 2009; Kodaira *et al.*, 2011). A number of genes have been reported to be induced or repressed in plants under water stress conditions (Yamaguchi-Shinozaki and Shinozaki, 2006; Nakashima *et al.* 2009; Golldack *et al.*, 2011; Lindemose *et al.*, 2013). Various transcription factors have been shown to be involved in ABA and water stress responses, such as those from the APETALA2/ethylene-responsive element-binding factor (AP2/ERF), NAM/ATAF1/CUC2 (NAC), WRKY, MYB, Cys2(C2)His2(H2)-type zinc finger protein (ZFP), basic helix–loop–helix (bHLH), and basic leucine zipper (bZIP) families (Yamaguchi- Shinozaki and Shinozaki, 2006; Nakashima *et al.* 2009; Fujita *et al.*, 2011; Golldack *et al.*, 2011; Lindemose *et al.*, 2013). These transcription factors function as transcriptional activators or repressors and control downstream gene expression in ABA and stress signal transduction pathways.

The Cys2/His2 (C2H2)-type zinc finger proteins (ZFPs), with 176 members in *Arabidopsis* and 189 members in rice, constitute one of the largest families of transcriptional regulators in plants (Agarwal *et al.*, 2007; Ciftci-Yilmaz and Mittler, 2008). It has been shown that C2H2-type ZFPs are important components in the regulation of plant growth, development, hormone responses, and tolerance to biotic and abiotic stresses (reviewed in Ciftci-Yilmaz and Mittler, 2008; Miller *et al.*, 2008; Kielbowicz-Matuk, 2012). In *Arabidopsis*, DNA chip analysis revealed that the transcript levels of several members of the C1-2i subclass (Zat family) in C2H2-type ZFPs were up-regulated by various biotic and abiotic stresses (Mittler *et al.*, 2006; Ciftci-Yilmaz and Mittler, 2008). Zat10 (STZ) and Zat12, two widely studied members of the Zat family, have been shown to be induced by drought, salinity, osmotic stress, temperature stress, wounding, oxidative stress, and high-light stress (Rizhsky *et al.*, 2004; Sakamoto *et al.*, 2004; Davletova *et al*., 2005*a*, *b*; Vogel *et al.*, 2005; Mittler *et al.*, 2006; Koussevitzky *et al.*, 2007; Rossel *et al.*, 2007; Ciftci-Yilmaz and Mittler, 2008; Miller *et al.*, 2008; Kielbowicz-Matuk, 2012). Genetic analysis revealed that *Zat10* and *Zat12* are required for the expression of ROS-scavenging genes and tolerance to drought, salinity, and oxidative stress (Rizhsky *et al.*, 2004; Sakamoto *et al.*, 2004; Mittler *et al.*, 2006; Rossel *et al.*, 2007; Ciftci-Yilmaz and Mittler, 2008; Miller *et al.*, 2008). In rice, several members of the C2H2-type ZFPs, such as ZFP182, ZFP245, ZFP252, and ZFP179, have also been shown to be involved in the responses of rice to drought, salinity, and oxidative stress (J. Huang *et al*., 2007, 2009, 2012; Xu *et al.*, 2008; Sun *et al.*, 2010). These results suggest that some members of the C2H2-type ZFPs are important regulators of ROS signalling under abiotic stresses. Until recently, however, it has not been clear whether these members of C2H2-type ZFPs are involved in ABA-induced antioxidant defence. By screening the homologous genes of *Zat12* in rice, an ABA- and H_2_O_2_-responsive ZFP gene, *ZFP182*, was identified in rice and was shown to be involved in ABA-induced antioxidant defence (Zhang *et al.*, 2012). In this study, by screening the homologous genes of *Zat10* in rice, a novel ABA- and H_2_O_2_-responsive C2H2-type ZFP gene, *ZFP36*, was identified in rice. By combining pharmacological, biochemical, and molecular biology analyses with genetic approaches, evidence is provided to show that ZFP36 plays a key role in ABA-induced antioxidant defence and tolerance of rice to water stress and oxidative stress. Moreover, it is revealed that ZFP36 is an important regulator of the cross-talk involving NADPH oxidase, H_2_O_2_, and MAPK in ABA signalling.

## Materials and methods

### Plant material and treatments

Seeds of rice (*Oryza sativa* L. sub. *japonica* cv. Nipponbare) were grown hydroponically with a nutrient solution in a light chamber at a temperature of 22 °C (night) to 28 °C (day), photosynthetic active radiation of 200 μmol m^–2^ s^–1^, and a photoperiod of 14/10h (day/night). When the second leaves were fully expanded, they were collected and used for investigations.

The plants were placed in beakers wrapped with aluminium foil with nutrient solution containing 100 μM ABA or 10mM H_2_O_2_ for the indicated time, with a continuous light intensity of 200 μmol m^–2^ s^–1^. To study the effects of inhibitors, the plants were pre-treated with 100 μM diphenylene iodonium (DPI) and 5mM dimethylthiourea (DMTU) for 2h, and then exposed to 100 μM ABA treatment under the same conditions as described above. Seedlings were treated with the nutrient solution alone under the same conditions for the whole period and served as controls for the above. After treatments of rice plants, the leaves were sampled and immediately frozen under liquid N_2_ for further analysis.

### Isolation of total RNA and semi-quantitative reverse transcription–polymerase chain reaction (RT–PCR)

Total RNA was isolated from leaves using RNAiso Reagent (TaKaRa, China) according to the manufacturer’s instructions. DNase treatment was included in the isolation step using RNase-free DNase (TaKaRa, China). Approximately 2 μg of total RNA were reverse transcribed using oligo d(T)_18_ primer and M-MLV reverse transcriptase (TaKaRa, China) at 42 °C for 90min and 75 °C for 15min. cDNA was amplified by PCR using the following primers: *ZFP36*, forward TTTTACGACGACGGCAACGG and reverse AGCGGCATCAGGTTGAGGTC; *OsMPK1*, forward AGAT ACATTCGCCAACTTCC and reverse TCTTAGAACAACACC TTCAGC; *OsMPK4*, forward CGCACAAACAACACTAAAGG and reverse GAGGTCCACAGGAAGAACAA; *OsMPK5*, forward GGGATCGTCTGCTCCGTGATGA and reverse AAGATGCAG CCGACGGACCA; *OsMPK7*, forward GGTGACTCCAGCCGA TA and reverse AGATACCTCCTTGCCTTGT; *OsMPK14*, forward TTGCTCTGCTTTGGACACTC and reverse CGTCTTGCC TTCTCATTTCTAA; and *GAPDH*, forward ACCACAAA CTGCCTTGCTCC and reverse ATGCTCGACCTGCTGTCACC. To standardize the results, the relative abundance of the glyceraldehyde-3-phosphate dehydrogenase gene (*GAPDH*) was also determined and used as the internal standard.

The cycle number of the PCRs was adjusted for each gene to obtain barely visible bands in agarose gels. Aliquots of the PCRs were loaded on agarose gels and stained with ethidium bromide.

### Cloning of *ZFP36*

According to the predicted sequence of *ZFP36*, two primers (forward 5′-CAATCCCATCAAATCATCCACCGC-3′ and reverse 5′-CACCAGCTTAGCTTGTTGCATACTCG-3′) were designed. The PCR conditions are as follows: 0.8 μl of reverse transcription product was amplified in a 20 μl volume containing 10 μl of 2×Taq Master Mix (Biodee, Nanjing, China), 0.4 μl of 10mM of each primer, and 8.4 μl of double-distilled water. PCR was performed on a DNA amplification machine (Dongsheng, China) for an initial denaturation at 94 °C for 5min; 35 cycles at 94 °C for 20 s, 58 °C for 20 s, 72 °C for 30 s; and a final step of 72 °C for 10min. The PCR products were run on an 1% agarose gel and purified with a Gel Extraction Kit (Generay, Shanghai, China) according to the manufacturer’s protocol. The purified product was then cloned into the pMD19-T vector (TaKaRa, China) and sequenced (BGI, Shenzhen, China).

### Real-time quantitative RT–PCR expression analysis

Real-time quantitative RT–PCRs were performed on a Bio-RAD MyiQ™ Real-time PCR Detection System (Bio-Rad, USA) using the SYBR^®^ Premix Ex Taq™ (TaKaRa, China) according to the manufacturer’s instructions. cDNA was amplified by PCR using the following primers: *ZFP36*, forward AACTAATTCATCATACGCCATC and reverse CAAAGAATAGACTCTGTTCAATAG; *OsMPK1*, forward CTGAAGGTGTTGTTCTAAGAGTAG and reverse TGATGAT AACCGCAGAATTTAGG; *OsMPK4*, forward AACCAAGGG AAGCATTACTAC and reverse GAGCAAACTATTCCATAAGCC; *OsMPK5*, forward CCGCTGCAGAGAATCACGTTG and reverse TCCTCGTTTAGAGCCTTCTGCTC; *OsMPK7*, forward TGTTT CTCTTCCAAGGGCTA and reverse ATCGGATTCCT CACCACC; *OsMPK14*, forward TGCTTTGGACACTCACACCG and reverse CCCCTTGATGGAGGAAGTAGAATA; *OsrbohB*, forward TGCTCTTTGTCCATGGAACGTG and reverse ACAG CGAGGTACATCCATGTCG; *OsrbohE*, forward TGGTCTTGG AATTGGTGCTACTCC and reverse ACCATGTATGCTTT CCACCTCTTC; *SodCc2*, forward GGAGAAGATGGTGTTGCTA and reverse GCCTTGAAGTCCGATGAT; *cAPX*, forward TGTCC TTGTCACTCAAACCCATC and reverse GACCAACTTCCCAT CCTCTCCTA; anbd *Osactin*, forward CTTCATAGGAAT GGAAGCTGCGGGTA and reverse CGACCACCTTGATCTT CATGCTGCTA. Each PCR (20 μl) contained 10 μl of 2× real-time PCR Mix (containing SYBR Green I), 0.4 μl of each primer, and appropriately diluted cDNA. The thermal cycling conditions were 95 °C for 30 s followed by 40 cycles of 95 °C for 10 s, 60 °C for 20 s, and 72 °C for 20 s. To standardize the data, the ratio of the absolute transcript level of the target genes to the absolute transcript level of *Osactin* was calculated for each sample. The relative expression levels of the target genes were calculated as x-fold changes relative to the appropriate control experiment for the different treatments.

### Protoplast isolation

Rice plants were grown in the dark at 28 °C for 1–2 weeks. When plants were ~4–8 inches tall, the protoplasts from the leaf and stem tissue were isolated according to the method described by Zhang *et al.* (2012).

### Double-stranded (ds) RNA synthesis and transfection

dsRNA was produced by *in vitro* transcription of a PCR-generated DNA template using the following primer pairs containing the T7 promoter sequence on both ends: dsZFP36, forward TAATACGACTCACTATAGGGAGACTA ATTCATCATACGCCATC and reverse TAATACGACTCACTATA GGGAGATGAATCAACACTCCTAGAACC (the underlined part indicates the T7 promoter sequence); dsMPK5, forward TAATA CGACTCACTATAGGGAGACCGCTGCAGAGAATCA CAGTTG and reverse TAATACGACTCACTATAGGGA GATCCTCGTTTAGAGCCTTCTGCTC; dsMPK1, forward TAA TACGACTCACTATAGGGAGAAGATACATTCGC CAACTTCC and reverse TAATACGACTCACTATAGGGA GATCTTAGAACAACACCTTCAGC; dsMPK4, forward TAATACGACTCACTATAGGGCCGCAAGCACATCCTCTT and reverse TAATACGACTCACTATAGGGCCTGCCACAT CATCTCCC; dsMPK7, forward TAATACGACTCACTA TAGGGTACGGTCTTCACAATACTACTT and reverse TAAT ACGACTCACTATAGGGATTCCCATCTTGCTCATC; and dsMPK14, forward TAATACGACTCACTATAGGGTAC GGTGAGGGAAACAGGT and reverse TAATACGACTCACTA TAGGGGTCGCAGGAGTCTAAGCAA. The PCR products were recovered and the concentrations were measured. dsRNAs were synthesized *in vitro* using the Promega Ribomax Large Scale RNA Production System T7 (Promega). The dsRNAs were purified by phenol–chloroform–isopropanol extraction, dissolved in RNase-free water, and quantified by UV spectrophotometry.

The dsRNAs were delivered into protoplasts using a polyethylene glycol (PEG)–calcium-mediated method described previously (Shi *et al.*, 2012).

### Generation of *ZFP36* transgenic rice

To obtain the transgenic plants with overexpression of *ZFP36*, the full-length open reading frame (ORF) of ZFP36 was inserted into the plant binary vector pCAMBIA1301 to construct pCAMBIA1301-*ZFP36*. Then the *ZFP36* gene under the control of the *Cauliflower mosaic virus* (CaMV) 35S promoter was transformed into rice (*O. sativa* sub. *japonica* cv. Nipponbare) by the *Agrobacterium*-mediated transformation method (Hiei *et al.*, 1994).

To generate *ZFP36-*RNAi (RNA interference) plants, the transcription region of the *ZFP36* gene (from +116bp to +366bp) was amplified with primers containing the following restriction enzyme sites: the 5′ most primer with *Nco*I and *Bam*HI sites, and the 3′-most primer with *Xho*I and *Spe*I sites. The resulting PCR product was digested first with *Nco*I and *Xho*I and ligated into a *Nco*I–*Xho*I-cleaved pGSA1285 vector (template plasmid). For the second PCR fragment for the inverted repeat construct, the same PCR product was digested with *Bam*HI and *Spe*I and inserted into the *Bam*HI–*Spe*I sites of the template plasmid. The *ZFP36*-RNAi plasmid was introduced into rice (*O. sativa* sub. *japonica* cv. Nipponbare) by the *Agrobacterium*-mediated transformation method (Hiei *et al.*, 1994).

### Tolerance of transgenic rice plants to PEG and H_2_O_2_ stresses

To evaluate the performance of the transgenic rice plants under abiotic stresses, the seeds of T_2_ transgenic lines (*ZFP36*-OE and *ZFP36*-RNAi) and the wild type (WT) were germinated and grown in a light chamber as described above. For the analysis of survival rate, the rice seedlings were treated with 18% PEG 4000, 100mM H_2_O_2_ for the indicated time, and the survival rates of the rice plants were counted after recovery by re-watering for 14 d. For the analysis of growth, the rice seedlings were treated with 18% PEG 4000, 100mM H_2_O_2_ for 15 d, and the shoot length and fresh weight, and root length were measured. For the analysis of oxidative damage to lipids and plasma membranes, the rice seedlings were treated with 15% PEG 4000, 100mM H_2_O_2_ for 2 d, and the content of malondialdehyde (MDA) and the percentage leakage of electrolyte were determined. For the analysis of antioxidant enzymes, the rice seedlings were treated with 15% PEG 4000, 100mM H_2_O_2_ for 12h, and the activities of superoxide dismutase (SOD) and ascorbate peroxidase (APX) were measured.

### Determination of lipid peroxidation and electrolyte leakage

Oxidative damage to lipids was estimated by measuring the content of MDA in leaf segment homogenates, prepared in 10% trichloroacetic acid containing 0.65% 2-thiobarbituric acid (TBA), and heated at 95 °C for 25min, as in Hodges *et al.* (1999). The percentage leakage of electrolyte was determined as described by Jiang and Zhang (2001).

### Enzyme assays

Frozen protoplasts or leaves were homogenized in a solution of 50mM potassium phosphate buffer (pH 7.0) containing 1mM EDTA and 1% polyvinylpyrrolidone. The homogenate was centrifuged at 12 000 *g* for 20min at 4 °C and the supernatant was immediately used for the antioxidant enzyme assays. The total activities of SOD and APX were determined as described previously (Jiang and Zhang, 2001).

### H_2_O_2_ detection by confocal laser scanning microscopy

H_2_O_2_ production in protoplasts was monitored using the H_2_O_2_-sensitive fluorescent probe 2′,7′-dichlorofluorescein diacetate (H_2_DCF-DA; Molecular Probes, Leiden, The Netherlands) using the method described by Bright *et al.* (2006). The specificity of the H_2_O_2_-mediated fluorescence was proved by the application of catalase (CAT) (Ding *et al.*, 2013). Images acquired were analysed using Leica IMAGE software (Leica Microsystems, Heerbrugge, Switzerland). Data are presented as mean pixel intensities. A total of 120 protoplasts are observed per treatment for three independent replicates.

### Determination of H_2_O_2_ content in leaf extracts

H_2_O_2_ from leaves was extracted according to a previously described method (Rao *et al.*, 2000). The content of H_2_O_2_ in leaf extracts was determined using the Hydrogen Peroxide Assay Kit (Beyotime Institute of Biotechnology, Shanghai, China) according to the manufacturer’s instructions. Briefly, test tubes containing 50 μl of supernatants and 100 μl of test solutions were placed at 30 °C for 30min and measured immediately with a spectrometer at a wavelength of 560nm. Absorbance values were calibrated to a standard curve generated with known concentrations of H_2_O_2_.

## Results

### Cloning and sequence analysis of *ZFP36*

**Fig. 1.**
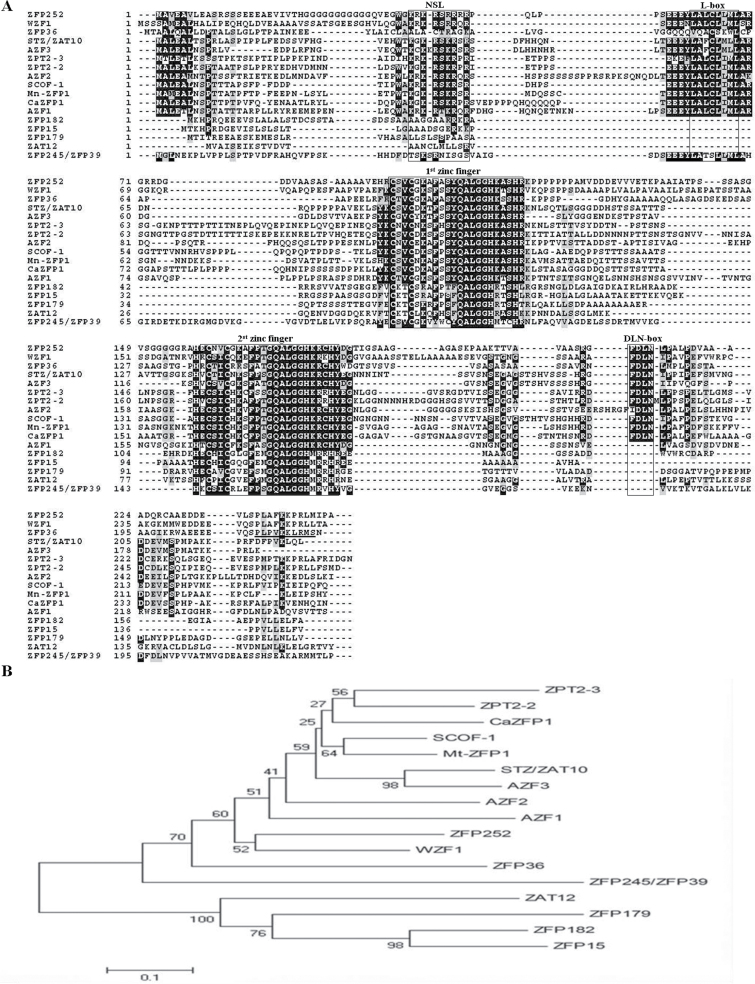
Alignment and phylogenetic relationship of ZFP36 with the other stress-responsive C2H2-type ZFPs. (A) Alignment of ZFP36 with other plant stress-responsive C2H2-type ZFPs. Characteristic amino acid sequences (NLS, L-box, two zinc fingers, and DLN-box) are boxed, and the putative NLS is underlined. Positions containing identical residues are shaded in black, while conservative residues are in grey. (B) The phylogenetic tree of plant stress-responsive C2H2-type ZFPs. Alignment of conserved ZFP sequences and phylogenetic analysis were performed by the program ClustalX 1.83 and MEGA 4, respectively. A distance tree was calculated using the Neighbor–Joining method. The lengths of the branches are proportional to the degree of divergence. Species designations and corresponding GenBank accession numbers are as follows: *A. thaliana*: AZF1 (BAA85108), AZF2 (BAB02542), AZF3 (AB030732), ZAT12 (AAM65582), STZ/ZAT10 (NP-174094); *C. annuum*: CaZFP1 (CAF74935); *G. max*: SCOF-1 (AAB39638); *M. truncatula*: Mt-ZFP1 (CAB77055); *O. sativa*: ZFP15 (AAP42460), ZFP36 (AAP51130.1), ZFP179 (AAL76091.1), ZFP182 (NP001051718.1), ZFP245 (AAQ95583), ZFP252 (AAO46041.1); *P. hybrida*: ZPT2-3 (BAA05079), ZPT2-2 (BAA05077.1); *T. aestivum*: WZF1 (BAA03902).

The *ZFP36* gene containing a complete ORF of 663bp was cloned by RT–PCR from total RNA prepared from rice seedlings. The predicted protein product of *ZFP36* comprises 220 amino acids with the calculated molecular mass of 22.804kDa. The ZFP36 protein contains two C2H2-type zinc fingers, with a plant-specific QALGGH motif in each zinc finger domain (Fig. 1A). A homology search against the GenBank database showed that ZFP36 was homologous to many C2H2-type ZFPs in plants, especially in the finger domains (Fig. 1A). Like most reported C2H2-type ZFPs, ZFP36 contains a DLN-box/EAR-motif with a consensus of DLN at the C-terminus and a putative nuclear localization signal (NLS) (Fig. 1A). Unlike most zinc finger proteins reported, however, the NLS of ZFP36 locates at the C-terminus.

To investigate the evolutionary relationship among plant C2H2-type ZFPs involved in stress responses, a phylogenetic tree was constructed using the Neighbor–Joining method with the full-length amino acid sequences (Fig. 1B). The result revealed that ZFP36 was clustered with *Arabidopsis* Zat10.

### ABA and H_2_O_2_ induce the expression of *ZFP36*, and H_2_O_2_ is required for the ABA-induced gene expression

**Fig. 2.**
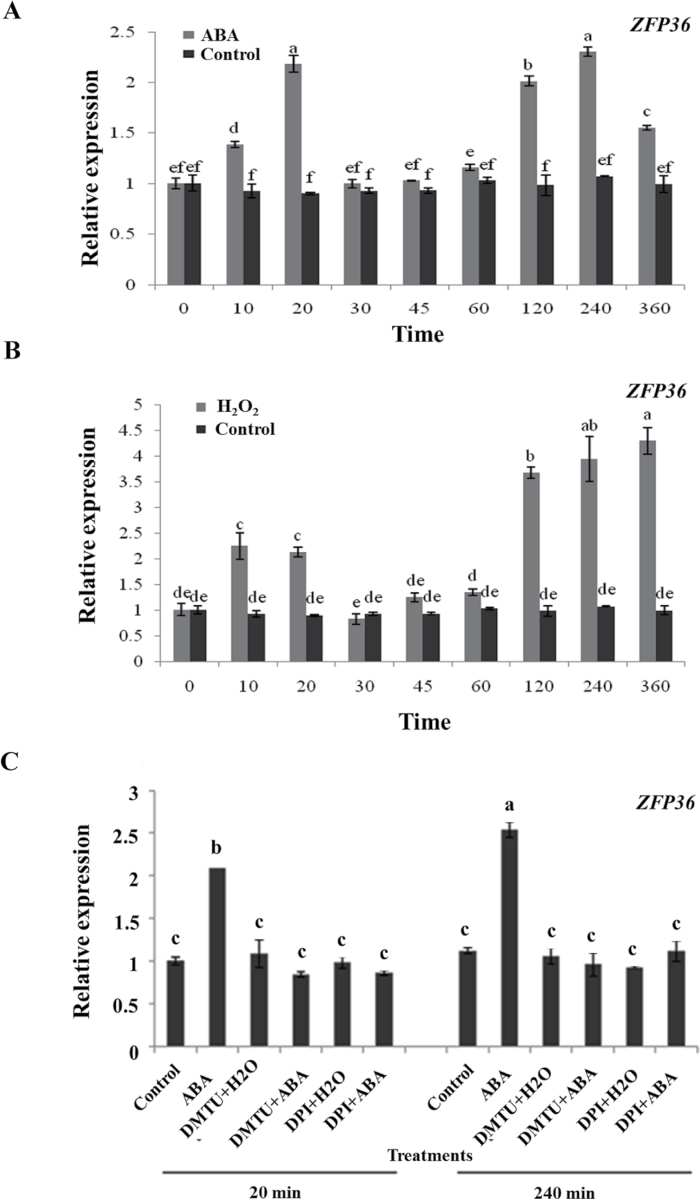
ABA and H_2_O_2_ induce the expression of *ZFP36* in rice leaves. (A) Expression analysis of *ZFP36* in leaves of rice plants exposed to ABA treatment. (B) Expression analysis of *ZFP36* in leaves of rice plants exposed to H_2_O_2_ treatment. (C) Effects of pre-treatments with dimethylthiourea (DMTU) and diphenylene iodonium (DPI) on the expression of *ZFP36* in rice leaves exposed to ABA treatment. In (A, B), the rice seedlings were treated with 100 μM ABA (A) or 10mM H_2_O_2_ (B) for various times as indicated. In (C), the rice seedlings were pre-treated with 5mM DMTU or 100 μM DPI for 2h, and then exposed to 100 μM ABA for 20min and 240min, respectively. Relative expression levels of the *ZFP36* gene were analysed by real-time quantitative RT–PCR. Values are means ±SE of three independent experiments. Means denoted by the same letter did not differ significantly at *P*<0.05 according to Duncan’s multiple range test.

To investigate the effects of ABA and H_2_O_2_ on the expression of *ZFP36* in leaves of rice seedlings, relative quantitative real-time PCR analysis was performed on total RNA isolated from rice leaves treated with ABA or H_2_O_2_. The results showed that the treatments with ABA and H_2_O_2_ induced a biphasic response in the expression of *ZFP36* in rice leaves (Fig. 2A, B). For ABA treatment, the first peak (phase I) in the expression of *ZFP36* occurred within 20min of ABA treatment, and the second peak (phase II) appeared after 4h of ABA treatment (Fig. 2A). For H_2_O_2_ treatment, phase I occurred within 10min of H_2_O_2_ treatment and phase II peaked after 6h of H_2_O_2_ treatment (Fig. 2B).

To establish a link between the production of H_2_O_2_ and the expression of *ZFP36* in ABA signalling, rice plants were pre-treated with the two ROS manipulators, DMTU, a scavenger for H_2_O_2_, and DPI, an inhibitor of NADPH oxidase, respectively, and then exposed to ABA treatment. Experimental results showed that pre-treatments with the two H_2_O_2_ manipulators dramatically abolished the phase I and phase II induced by ABA (Fig. 2C), suggesting that H_2_O_2_ is required for ABA-induced up-regulation in the expression of *ZFP36*.

### *ZFP36* is involved in ABA-induced antioxidant defence and enhances the tolerance of rice to water stress and oxidative stress

**Fig. 3.**
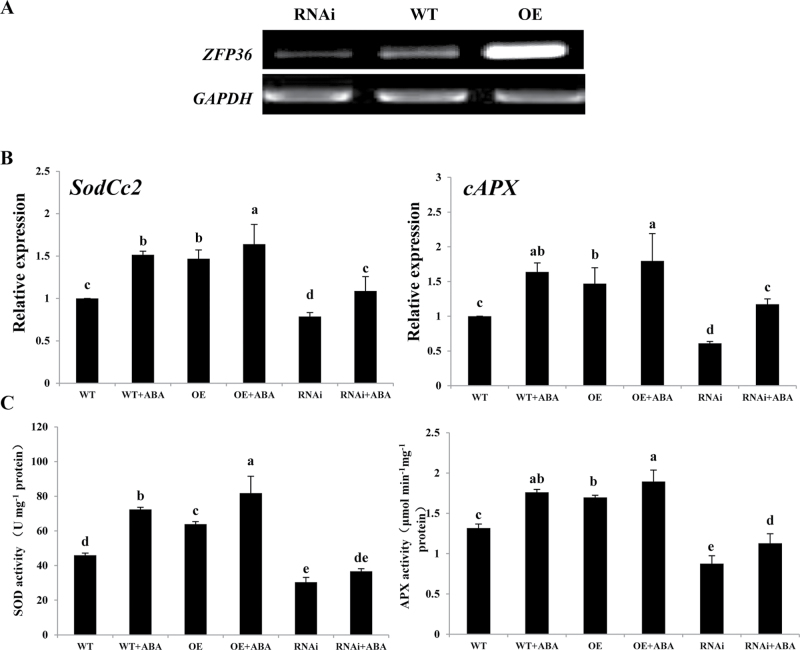
ZFP36 is required for ABA-induced up-regulation of the expression and the activities of antioxidant enzymes in rice leaves. (A) The expression of *ZFP36* analysed by RT–PCR in *ZFP36*-OE plants, *ZFP36*-RNAi plantsm and WT plants grown under control conditions. The glyceraldehyde-3-phosphate dehydrogenase gene (*GAPDH*) was amplified as a control for the amount of template. (B, C) The expression of *SodCc2* and *cAPX* (B) and the activities of SOD and APX (C) in the leaves of *ZFP36*-OE plants, *ZFP36*-RNAi plants, and WT plants. The rice seedlings were treated with 100 μM ABA for 12h, and the relative expression levels of *SodCc2* and *cAPX* and the activities of SOD and APX were analysed. In (A), experiments were repeated at least three times with similar results. In (B, C), values are means ±SE of three independent experiments. Means denoted by the same letter did not differ significantly at *P*<0.05 according to Duncan’s multiple range test.

To investigate whether *ZFP36* is involved in ABA-induced antioxidant defence, *ZFP36*-overexpressing (*ZFP36*-OE) and silenced (*ZFP36*-RNAi) transgenic rice plants were generated. As shown in Fig. 3A, the expression of *ZFP36* in *ZFP36*-OE plants was significantly higher than in WT plants, and the expression of *ZFP36* in *ZFP36*-RNAi plants was obviously lower than that in WT plants. Under the control conditions, the expression of the antixiodant genes *SodCc2*, encoding a cytosolic Cu/Zn-SOD, and *cAPX*, encoding a cytosolic APX, and the activities of SOD and APX were higher in the leaves of the *ZFP36*-OE plants than in those in the WT plants, and the expression and the activities of these antioxidant enzymes were lower in the *ZFP36*-RNAi plants than in the WT plants (Fig. 3B, C). ABA treatment induced significant increases in the expression of *SodCc2* and *cAPX* and the activities of SOD and APX in leaves of WT plants, and the increases induced by ABA were further enhanced in the leaves of the *ZFP36*-OE plants, but were inhibited in the *ZFP36*-RNAi plants (Fig. 3B, C). These results indicate that ZFP36 is required for ABA-induced increases in the expression and the activities of SOD and APX.

**Fig. 4.**
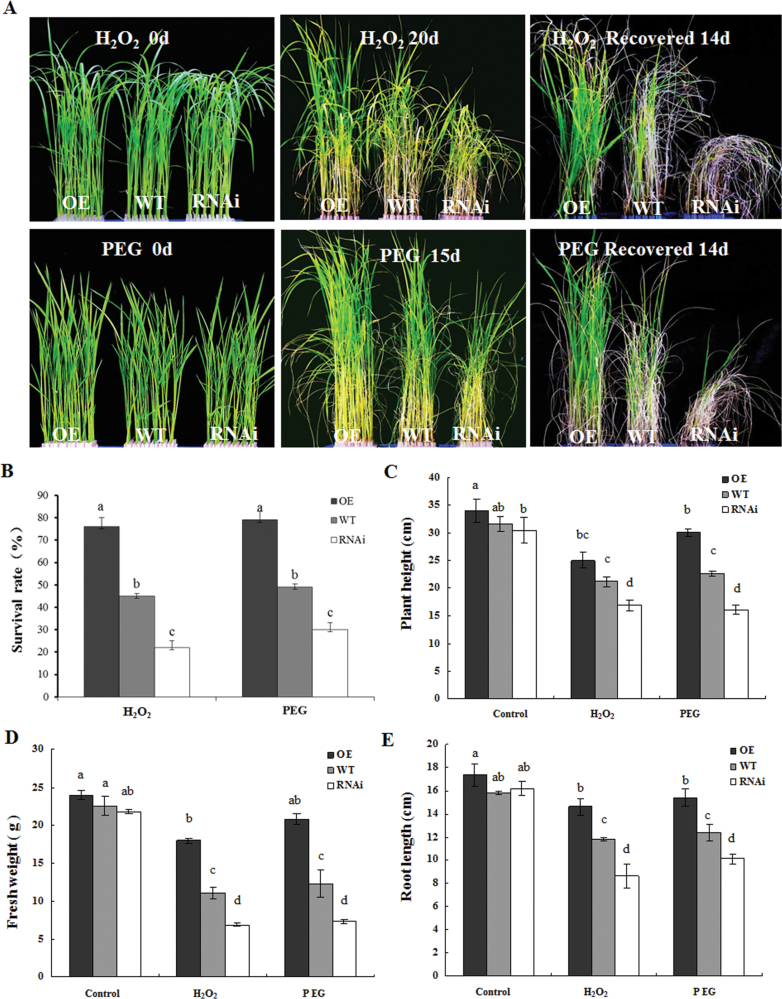
*ZFP36* enhances the tolerance of rice plants to water stress and oxidative stress. (A) Photographs of *ZFP36*-OE plants, *ZFP36*-RNAi plants, and WT plants grown under water stress and oxidative stress. Ten-day-old rice seedlings were treated with 18% PEG 4000 for 15 d or 2-week-old plants were treated with 100mM H_2_O_2_ for 20 d, and then left to recover for 14 d. (B) The survival rate (%) of the rice plants after recovery by re-watering for 14 d shown in (A). (C–E) The growth analysis of *ZFP36*-OE plants, *ZFP36*-RNAi plants, and WT plants under water stress and oxidative stress. Ten-day-old rice seedlings were treated with 18% PEG 4000, 100mM H_2_O_2_ for 15 d, and the lengths of shoots (C) and roots (E) and shoot fresh weight (D) were measured. In (A), experiments were repeated at least three times with similar results. In (B–E), values are means ±SE of three independent experiments. Means denoted by the same letter did not differ significantly at *P*<0.05 according to Duncan’s multiple range test.

To test the role of ZFP36 in the tolerance of water stress and oxidative stress in plants, the rice seedlings of the WT, *ZFP36*-OE, and *ZFP36*-RNAi were treated with PEG (simulation of water stress) and H_2_O_2_. Under the control conditions, there were no significant differences in plant height (Fig. 4A, C), shoot fresh weight (Fig. 4D), and root length (Fig. 4E) between the *ZFP36*-OE line or *ZFP36*-RNAi line and WT plants. Under water stress induced by 18% PEG 4000 and oxidative stress induced by 100mM H_2_O_2_, however, the *ZFP36*-OE plants had longer shoot and root lengths, greater fresh weight, and higher survival rate than WT plants (Fig. 4A–E). In contrast, the *ZFP36*-RNAi plants exhibited shorter shoot and root lengths, lower fresh weight, and lower survival rate than WT plants under the stressed conditions (Fig. 4A–E). These results indicate that ZFP36 is required for the tolerance of rice to water stress and oxidative stress.

**Fig. 5.**
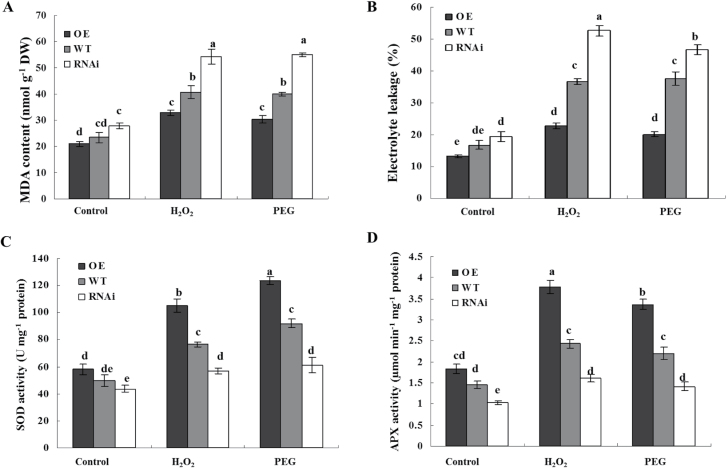
*ZFP36* up-regulates the activities of antioxidant enzymes and reduces oxidative damage under water stress and oxidative stress. (A, B) The content of MDA (A) and the percentage leakage of electrolyte (B) in the leaves of *ZFP36*-OE plants, *ZFP36*-RNAi plants, and WT plants. Ten-day-old rice seedlings were treated with 15% PEG 4000, 100mM H_2_O_2_ for 2 d, and then leaves were sampled for the determination of MDA content and electrolyte leakage (%).(C, D) The activities of SOD (C) and APX (D) in the leaves of *ZFP36*-OE plants, *ZFP36*-RNAi plants, and WT plants. Ten-day-old rice seedlings were treated with 15% PEG 4000, 100mM H_2_O_2_ for 12h, and then leaves were sampled for the determination of the activities of SOD and APX. Values are means ±SE of three independent experiments. Means denoted by the same letter did not differ significantly at *P*<0.05 according to Duncan’s multiple range test.

To investigate the role of ZFP36 in antioxidant defence under stressed conditions, the content of MDA and the percentage of electrolyte leakage, which are indications of oxidative stress, and the activities of the antioxidant enzymes SOD and APX were analysed in gain- and loss-of-fuction *ZFP36* rice plants exposed to water stress and oxidative stress. The content of MDA (Fig. 5A) and the percentage of electrolyte leakage (Fig. 5B) were lower in the leaves of the *ZFP36*-OE plants exposed to 15% PEG and 100mM H_2_O_2_ treatment than in those of the WT plants, and the activities of SOD (Fig. 5C) and APX (Fig. 5D) in the *ZFP36*-OE plants were higher than the WT plants under the stressed conditions. In contrast, under water stress and oxidative stress, the content of MDA (Fig. 5A) and the percentage of electrolyte leakage (Fig. 5B) were higher in the leaves of the *ZFP36*-RNAi plants than in those of the WT plants, and the activities of SOD (Fig. 5C) and APX (Fig. 5D) in the *ZFP36*-RNAi plants were lower than in the WT plants. These results suggest that the expression of *ZFP36* can enhance the ability of rice plants to scavenge ROS, thus resulting in the reduction of oxidative damage caused by water stress and oxidative stress.

### *ZFP36* regulates the expression of NADPH oxidase and MAPK genes and the production of H_2_O_2_ in ABA signalling

**Fig. 6.**
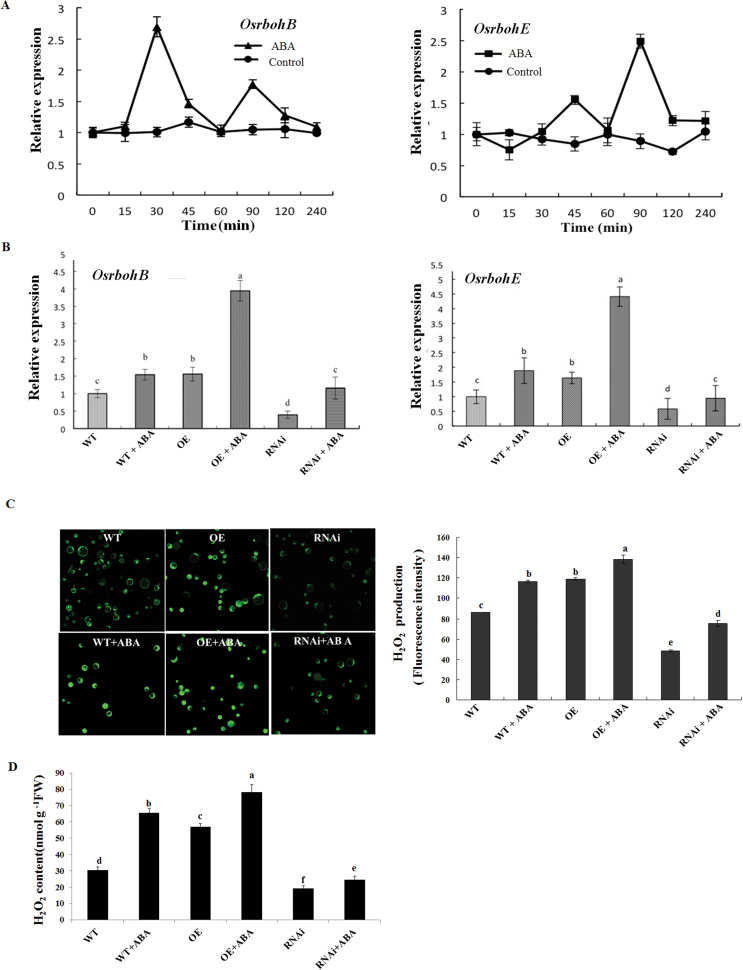
*ZFP36* regulates the expression of NADPH oxidase genes and the production of H_2_O_2_ in ABA signalling. (A) Time course of changes in the expression of *OsrbohB* and *OsrbohE* in leaves of rice plants exposed to ABA treatment. The rice seedlings were treated with 100 μM ABA for various times as indicated, and the relative expression levels of *OsrbohB* and *OsrbohE* were analysed by real-time quantitative RT–PCR. (B) The expression of *OsrbohB* and *OsrbohE* in the leaves of *ZFP36*-OE plants, *ZFP36*-RNAi plants, and WT plants. The rice seedlings were treated with 100 μM ABA for 30min (*OsrbohB*) or 90min (*OsrbohE*), and the relative expression levels of *OsrbohB* and *OsrbohE* were analysed by real-time quantitative RT–PCR. (C) The production of H_2_O_2_ in the protoplasts from *ZFP36*-OE plants, *ZFP36*-RNAi plants, and WT plants. The protoplasts were treated with 10 μM ABA (+ABA) or the incubation medium (–ABA) for 5min, and then loaded with H_2_DCF-DA for 10min. H_2_O_2_ was visualized by confocal microscopy (left), and the fluorescence intensity was analysed by Leica IMAGE software (right). (D) The content of H_2_O_2_ in the leaves of *ZFP36*-OE plants, *ZFP36*-RNAi plants, and WT plants. The rice seedlings were treated with 100 μM ABA for 2h, and the content of H_2_O_2_ in leaves was analysed. Values are means ±SE of three independent experiments. Means denoted by the same letter did not differ significantly at *P*<0.05 according to Duncan’s multiple range test.

Previous studies have shown that NADPH oxidase, H_2_O_2_, and MAPK are important components in ABA-induced antioxidant defence and that there is a positive feedback loop involving NADPH oxidase, H_2_O_2_, and MAPK in ABA signalling (Zhang *et al.*, 2006; Lin *et al.*, 2009). To investigate whether *ZFP36* regulates the expression of NADPH oxidase genes and the production of H_2_O_2_ in ABA signalling, *ZFP36*-OE and *ZFP36*-RNAi transgenic rice plants and WT plants were used. In the rice genome, there are nine NADPH oxidase (rboh) genes (*OsrbohA–OsrbohI*; Wong *et al.*, 2007). ABA treatment only induced increased expression of *OsrbohB* and *OsrbohE* in leaves of rice plants (Fig. 6A; data not shown). Under the control conditions, the expression of *OsrbohB* and *OsrbohE* was higher in the leaves of the *ZFP36*-OE plants than that in the WT plants, and the expression of these genes was lower in the *ZFP36*-RNAi plants than in the WT (Fig. 6B). ABA treatment induced significant increases in the expression of *OsrbohB* and *OsrbohE* in leaves of WT plants, and the increases were further enhanced in the leaves of the *ZFP36*-OE plants, but were inhibited in the *ZFP36*-RNAi plants (Fig. 6B). The changes in the production of H_2_O_2_, detected by confocal laser scanning microscopy in rice protoplasts (Fig. 6C) and by spectrophotometry in leaf extracts (Fig. 6D), in the transgenic rice plants and WT plants with or without ABA were similar to the changes in the expression of *OsrbohB* and *OsrbohE* (Fig. 6B).

**Fig. 7.**
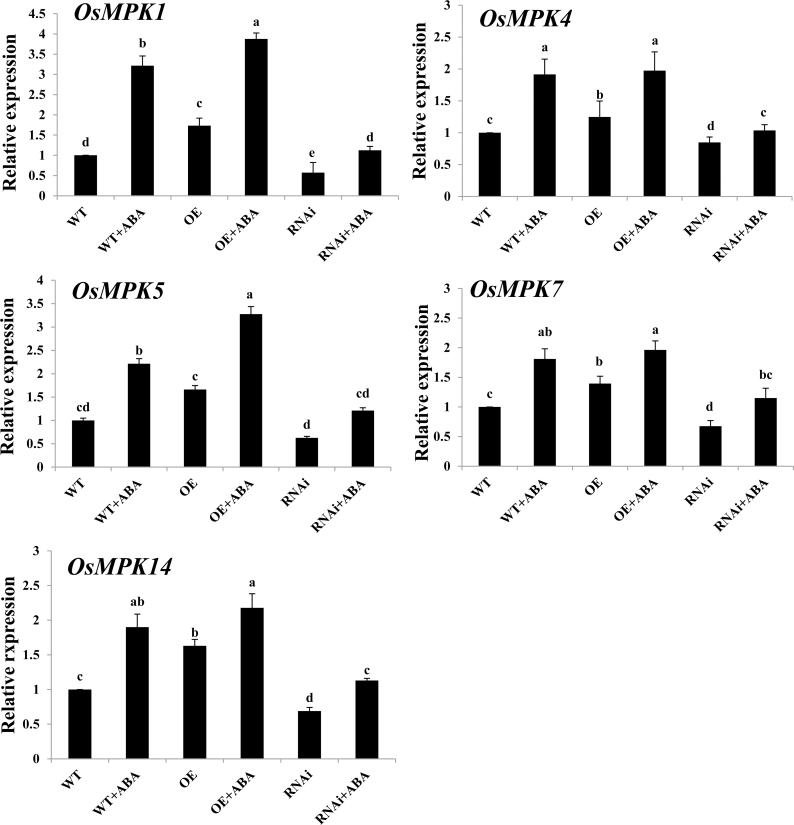
The expression of *OsMPK* genes in leaves of *ZFP36*-OE plants, *ZFP36*-RNAi plants, and WT plants. The rice seedlings were treated with 100 μM ABA for 30min (for *OsMPK4*, *OsMPK5*, *OsMPK7*, and *OsMPK14*) or 60min (for *OsMPK1*), and the relative expression levels of *OsMPK* genes were analysed by real-time quantitative RT–PCR. Values are means ±SE of three independent experiments. Means denoted by the same letter did not differ significantly at *P*<0.05 according to Duncan’s multiple range test.

In the rice genome, there are 17 MAPK genes (*OsMPK* genes; Reyna and Yang, 2006). Previous studies have shown that OsMPK1 and OsMPK5 are important components of ABA signalling (Zhang *et al.*, 2012; Shi *et al.*, 2014). Here another three MAPK genes were identified, *OsMPK4*, *OsMPK7*, and *OsMPK14*, which were also induced by ABA treatment (Supplementary Fig. S1 available at *JXB* online). To investigate whether *ZFP36* regulates the expression of these MAPK genes in ABA signalling, *ZFP36*-OE and *ZFP36*-RNAi transgenic rice plants and WT plants were used. The changes in the expression of *OsMPK* genes in the transgenic rice plants and WT plants with or without ABA (Fig. 7) were similar to those in the expression of *OsrbohB* and *OsrbohE* (Fig. 6B) and the production of H_2_O_2_ (Fig. 6C, D).

### The expression of *ZFP36* is regulated by OsMPKs in ABA signalling

**Fig. 8.**
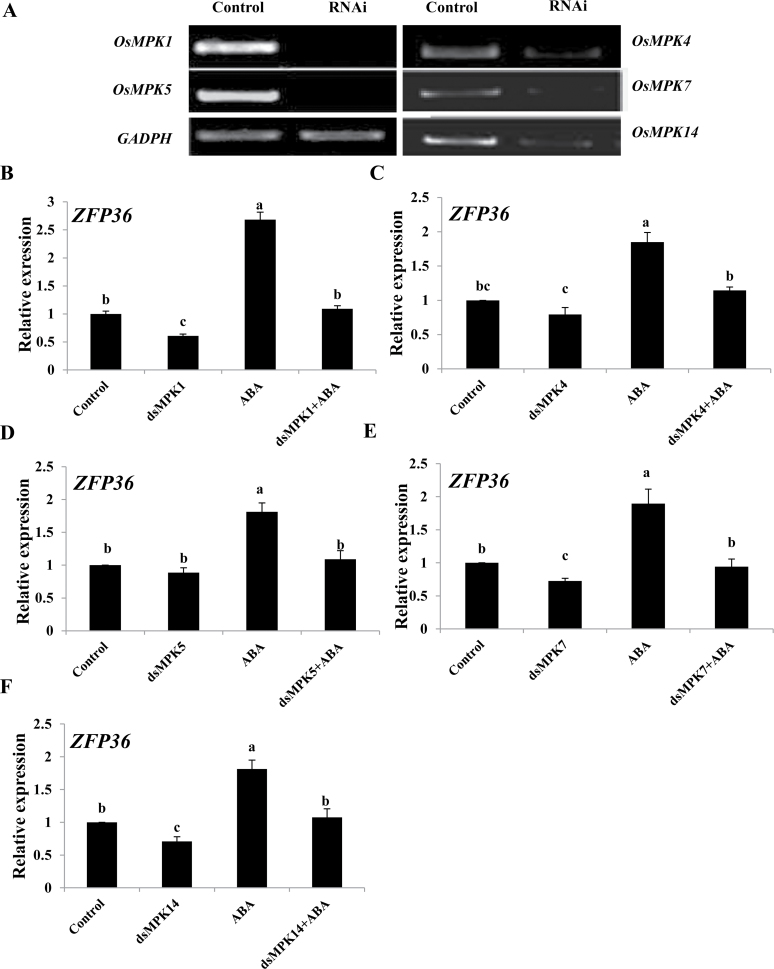
*OsMPK* genes regulate the expression of *ZFP36* in rice protoplasts. (A) The transient RNA interference (RNAi) silencing of *OsMPK1*, *OsMPK4*, *OsMPK5*, *OsMPK7*, and *OsMPK14* in rice protoplasts. The protoplasts were transfected with dsRNAs against *OsMPK* genes or with water (control) and incubated for 24h. The transient RNAi silencing of *OsMPK* genes was analysed by semi-quantitative RT–PCR. The glyceraldehyde-3-phosphate dehydrogenase gene (*GAPDH*) was amplified as a control for amount of a template. (B–F) The transient RNAi silencing of *OsMPK1* (B), *OsMPK4* (C), *OsMPK5* (D), *OsMPK7* (E), and *OsMPK14* (F) reduces the ABA-induced expression of *ZFP36* in rice protoplasts. The protoplasts were treated with 10 μM ABA for 5min, and the relative expression levels of *ZFP36* were analysed by real-time quantitative RT–PCR. In (A), experiments were repeated at least three times with similar results. In (B–F), values are means ±SE of three independent experiments. Means denoted by the same letter did not differ significantly at *P*<0.05 according to Duncan’s multiple range test.

To investigate whether OsMPKs are also involved in the ABA-induced up-regulation of the expression of *ZFP36*, a transient RNAi analysis in protoplasts (Zhai *et al.*, 2009), which has been proven to be gene specific (Zhai *et al.* 2009; Zhang *et al.* 2012; Cao *et al.* 2014), was used. In rice protoplasts, RNAi-mediated silencing of *OsMPK1*, *OsMPK4*, *OsMPK5*, *OsMPK7*, and *OsMPK14* substantially decreased the expression of these genes (Fig. 8A), and also decreased the expression of *ZFP36* (Fig. 8B–F). Further, the ABA-induced increase in the expression of *ZFP36* in the control protoplasts was inhibited by the RNAi silencing of these *OsMPK* genes (Fig. 8B–F). These results indicate that these OsMPKs are required for ABA-induced up-regulation in the expression of *ZFP36*.

## Discussion

Recent studies have revealed that some members of C2H2-type ZFPs in *Arabidopsis*, such as Zat10, Zat12, Zat7, Zat6, AZF1, AZF2, and AZF3, are involved in the responses of plants to various abiotic stresses (Ciftci-Yilmaz and Mittler, 2008; Miller *et al.*, 2008; Kielbowicz-Matuk, 2012). Zat10 and Zat12, the two widely studied members of the Zat family, have been shown to be induced by multiple abiotic stresses and oxidative stress (Rizhsky *et al.*, 2004; Sakamoto *et al.*, 2004; Davletova *et al*., 2005*a*, *b*; Vogel *et al.*, 2005; Mittler *et al.*, 2006; Koussevitzky *et al.*, 2007; Rossel *et al.*, 2007; Ciftci-Yilmaz and Mittler, 2008; Miller *et al.*, 2008; Kielbowicz-Matuk, 2012). *Zat12*-overexpressing *Arabidopsis* plants showed higher tolerance to cold, oxidative, osmotic, and high-light stresses, and knockout *Zat12 Arabidopsis* seedlings suffered increased sensitivity to these stresses (Iida *et al.*, 2000; Rizhsky *et al.*, 2004; Davletova *et al.*, 2005*b*; Vogel *et al.* 2005). Similarly, transgenic plants overexpressing *Zat10* were found to be more tolerant to drought, salinity, osmotic stress, heat stress, and high-light stress (Sakamoto *et al.*, 2004; Mittler *et al.*, 2006; Rossel *et al.*, 2007), but, surprisingly, *Zat10* knockout and RNAi lines were also more tolerant to osmotic stress and salinity (Mittler *et al.*, 2006). These results suggest that the role of these ZFPs in tolerance of plants to abiotic stresses is complex. This is further supported by AZF1 and AZF2, which are closely related to Zat10 in structure, and were found to regulate salt tolerance negatively in *Arabidopsis* (Kodaira *et al.*, 2011). In rice, overexpression of the C2H2-type ZFP genes, *ZFP252*, *ZFP245*, and *ZFP179*, increased the tolerance of rice seedlings to drought, salinity, cold, and oxidative stress (Xu *et al.*, 2008; J. Huang *et al.*, 2009; Sun *et al.*, 2010). However, DST (drought and salt tolerance), a C2H2-type ZFP in rice, negatively regulates rice tolerance to drought and salt stresses (X.Y. Huang *et al.*, 2009). In the present study, evidence is provided to show that ZFP36, a novel rice C2H2-type ZFP, is a positive regulator of rice tolerance to water stress and oxidative stress. Transgenic rice plants with overexpression of *ZFP36* showed a better phenotype in the lengths of the shoot and root, the shoot fresh weight, and the survival rate under water stress and oxidative stress than the WT plants, and the *ZFP36*-RNAi plants were more sensitive to water stress and oxidative stress than the WT (Fig. 4). These results indicate that ZFP36 is an important player in the response of rice to water stress and oxidative stress.

It has been shown that Zat10- and Zat12-dependent increased tolerance to abiotic stresses is associated with the up-regulation of antioxidant defence systems. *APX1*, *APX2*, and *FeSOD1* transcripts were constantly elevated in *Zat10* transgenic plants and showed suppressed expression in knockout *Zat10* plants during high-light stress (Mittler *et al.*, 2006). *Zat12* was shown to be required for *APX1* expression during oxidative stress (Davletova *et al.*, 2005*a*), although overexpression of *Zat12* did not induce the expression of *APX1* (Davletova *et al.*, 2005*b*). In rice, overexpression of *ZFP245* and *ZFP179* enhanced the activities of SOD and peroxidase (POD) under drought, cold, and salt stresses, and increased the tolerance of rice seedlings to oxidative stress (J. Huang *et al.*, 2009; Sun *et al.*, 2010). DST was shown to bind directly to the promoter sequence of *peroxidase 24 precursor* to regulate the expression of the gene, thus resulting in the reduction of H_2_O_2_ accumulation (X.Y. Huang *et al.*, 2009). In the present study, the results showed that under the control conditions, the activities of SOD and APX were enhanced in *ZFP36*-OE transgenic plants and were decreased in *ZFP36*-RNAi plants (Fig. 5). Under water stress and oxidative stress, higher activities of SOD and APX were accompanied by lower oxidative damage in *ZFP36*-OE transgenic plants, and lower activities of SOD and APX were accompanied by higher oxidative damage in *ZFP36*-RNAi plants (Fig. 5). These results suggest that ZFP36-enhanced tolerance of rice plants to water stress and oxidative stress is related to the increase in the activities of antioxidant enzymes.

ABA, as a stress hormone, plays a key role in the regulation of plant water balance and water stress tolerance under drought and salt stress conditions (Cutler *et al.*, 2010; Hubbard *et al.*, 2010; Umezawa *et al.*, 2010; Fujita *et al.*, 2011). Accumulating evidence indicates that ABA-enhanced water stress tolerance results, at least in part, from the induction of antioxidant defence systems (Jiang and Zhang, 2002*a*, *b*, 2004; Miao *et al.*, 2006; Zhang *et al.*, 2006, 2007; Xing *et al*., 2007, 2008; Neill *et al.*, 2008; Miller *et al.*, 2010; Ye *et al.*, 2011). It was also shown that ABA treatment induced the expression of *Zat10*, *Zat12*, *AZF1*, and *AZF2* in *Arabidopsis* (Sakamoto *et al.* 2004; Davletova *et al.* 2005*b*; Kodaira *et al.* 2011; Kielbowicz-Matuk, 2012), and *ZFP182* and *ZFP179* in rice (Huang *et al.* 2007; Sun *et al.* 2010; Zhang *et al.*, 2012), suggesting that there might be a link between ABA-induced ZFPs and ABA-induced antioxidant defence under water stress. However, overexpression of *AZF1* and *AZF2* did not alter the expression of *APX1*, *APX2*, and *FeSOD1* in *Arabidopsis* (Kodaira *et al.* 2011). DST was shown to regulate stomatal closure negatively by direct modulation of genes related to H_2_O_2_ homeostasis, but the pathway was ABA independent (X.Y. Huang *et al.*, 2009). Using a transient gene expression analysis and a transient RNAi analysis in protoplasts, a recent study showed that ZFP182 is involved in ABA-induced up-regulation of the activities of antioxidant enzymes in rice (Zhang *et al.*, 2012). Here, another rice C2H2-type ZFP, ZFP36, is reported which is also involved in the regulation of antioxidant defence in ABA signalling. Treatments with ABA and H_2_O_2_ induced the expression of *ZFP36* (Fig. 2), and H_2_O_2_ is required for the ABA-induced up-regulation of the expression of *ZFP36* (Fig. 2). Overexpression of *ZFP36* in transgenic rice plants enhanced the expression of the antioxidant genes *SodCc2* and *cAPX* and the activities of SOD and APX, and RNAi silencing of *ZFP36* in transgenic plants decreased the expression of *SodCc2* and *cAPX* and the activities of SOD and APX (Figs 3, 5). Further, ABA-induced increases in the expression of *SodCc2* and *cAPX* and the activities of SOD and APX were further enhanced in the leaves of the *ZFP36*-OE plants, but were inhibited in the *ZFP36*-RNAi plants (Fig. 3). These results indicate that ZFP36 is required for ABA-induced up-regulation in the expression and the activities of antioxidant enzymes.

Previous studies have shown that NADPH oxidase, H_2_O_2_, and MAPK are important players in ABA-induced antioxidant defence and that there is a cross-talk between NADPH oxidase, H_2_O_2_, and MAPK in ABA signalling (Zhang *et al.*, 2006; Lin *et al.*, 2009). ABA-induced H_2_O_2_ production activates MAPKs, which amplify the H_2_O_2_ signal by regulating the activity of NADPH oxidase in ABA signalling. However, it is not clear whether C2H2-type ZFPs are involved in the regulation of the cross-talk in ABA signalling. It was reported that the expression of *ZFP182* was regulated by *OsMPK1* and *OsMPK5* in ABA signalling (Zhang *et al.*, 2012). However, *ZFP182* did not regulate the expression of *OsMPK1* and *OsMPK5* induced by ABA. These results suggest that ZFP182 might not be involved in the regulation of the cross-talk involving NADPH oxidase, H_2_O_2_, and MAPK in ABA signalling. In the present study, it is shown that ZFP36 is a key component in the regulation of the cross-talk in ABA signalling. On the one hand, the expression of *ZFP36* was regulated by H_2_O_2_, *OsMPK1*, *OsMPK4*, *OsMPK5*, *OsMPK7*, and *OsMPK14* in ABA signalling (Figs 2, 8), and, on the other hand, *ZFP36* regulated the expression of the NADPH oxidase genes *OsrbohB* and *OsrbohE* and the production of H_2_O_2_, and the expression of *OsMPK1*, *OsMPK4*, *OsMPK5*, *OsMPK7*, and *OsMPK14* in ABA signalling (Figs 6, 7). These results suggest that ZFP36 is a key regulator of ROS signalling in ABA signal transduction. However, it may be asked where the early H_2_O_2_ source for the expression of *ZFP36* in ABA signalling comes from. In *Arabidopsis*, it was shown that NADPH oxidases mediate ABA-induced ROS production in guard cells, and AtrbohD and AtrbohF are the major catalytic subunits in this process (Kwak *et al.*, 2003). SNF1-related protein kinase 2 (SnRK2) has been shown to be a positive regulator of the early ABA signalling module (Hubbard *et al.*, 2010; Umezawa *et al.*, 2010). OST1 (Open Stomata 1), a member of the SnRK2 family, was shown to be involved in ABA-induced ROS production, and to function upstream of ROS production (Mustilli *et al.*, 2002). A recent study further showed that AtrbohF interacts with and is phosphorylated by OST1, suggesting that OST1 regulates AtrbohF activity (Sirichandra *et al.*, 2009). However, the physiological importance of the phosphorylation of AtrbohF by OST1 in ABA signalling remains to be determined.

The mechanisms by which ZFP36 regulates the expression of these genes involved in ABA signalling are still undetermined. ZFP36 contains two typical C2H2 zinc finger domains and a DLN-box/EAR-motif located at its C-terminus. Several zinc finger proteins containing a DLN-box/EAR-motif have exhibited transcription-repressive activities in plants, such as petunia ZPT2-3 (Sugano *et al.*, 2003) and *Arabidopsis* Zat7 (Ciftci-Yilmaz *et al.*, 2007), STZ/Zat10, AZF1, and AZF2 (Sakamoto *et al.*, 2004). Overexpression of *AZF1* and *AZF2* in *Arabidopsis* repressed the expression of various genes, including osmotic stress and ABA-repressive genes and auxin-inducible genes (Kodaira *et al.*, 2011). However, the rice ZFP179 (Sun *et al.*, 2010), the chrysanthemum CgZFP1 (Gao *et al.*, 2012), the salt cress ThZF1 (Xu *et al.*, 2007), and the chickpea CaZF (Jain *et al.*, 2009) also have a DLN-box/EAR-motif in their C-terminal end and function as transcriptional activators in yeast. Moreover, both *Zat10* overexpressors and loss-of-function mutants display enhanced tolerance to drought and salinity stresses, suggesting that Zat10 plays a dual role as both an activator and a repressor of stress response genes (Mittler *et al.*, 2006). These contrasting data suggest the existence of complex networks of interactions between ZFPs and different partners to regulate the tolerance of plants to water stress and oxidative stress. The exact mechanisms of how ZFP36 up-regulates the expression of the ABA-inducible genes in ABA signalling are under investigation in the authors’ laboratory.

In conclusion, the present data indicate that ZFP36 is required for ABA-induced antioxidant defence and for tolerance of rice plants to water stress and oxidative stress. ABA-induced H_2_O_2_ production and ABA-induced activation of OsMPKs up-regulate the expression of *ZFP36* in ABA signalling, and the expression of *ZFP36* also up-regulates the expression of NADPH oxidase and MAPK genes and the production of H_2_O_2_ in ABA signalling, indicating that ZFP36 is an important player in the regulation of the cross-talk involving NADPH oxidase, H_2_O_2_, and MAPK in ABA signalling. The results also suggest that ZFP36 is a key regulator of ROS signalling in the signal transduction of ABA, water stress, and oxidative stress.

## Supplementary data

Supplementary data are available at *JXB* online

Figure S1. Time courses of changes in the expression of *OsMPK4*, *OsMPK7*, and *OsMPK14* in response to ABA treatment.

Supplementary Data
