# Supplementary material for: A novel rice C2H2-type zinc finger protein, ZFP36, is a key player involved in abscisic acid-induced antioxidant defence and oxidative stress tolerance in rice
Source: J Exp Bot. 2014 Jul 28;65(20):5795–809. doi: 10.1093/jxb/eru313 (PMC4203119; doi:10.1093/jxb/eru313)
Supplement: Supplementary Data [file supp_65_20_5795__index.html]

A novel rice C2H2-type zinc finger protein, ZFP36, is a key player involved in abscisic acid-induced antioxidant defence and oxidative stress tolerance in rice — A novel rice C2H2-type zinc finger protein, ZFP36, is a key player involved in abscisic acid-induced antioxidant defence and oxidative stress tolerance in rice — Supplementary Data 

# A novel rice C2H2-type zinc finger protein, ZFP36, is a key player involved in abscisic acid-induced antioxidant defence and oxidative stress tolerance in rice

## Supplementary Data

Data files

**Files in this Data Supplement:**

- Supplementary Data - Supplementary Data
