# Supplementary material for: A novel rice C2H2-type zinc finger protein, ZFP36, is a key player involved in abscisic acid-induced antioxidant defence and oxidative stress tolerance in rice
Source: J Exp Bot. 2014 Jul 28;65(20):5795–809. doi: 10.1093/jxb/eru313 (PMC4203119; doi:10.1093/jxb/eru313)

A novel rice C2H2-type zinc finger protein, ZFP36, is a key player involved in abscisic acid-induced antioxidant defence and oxidative stress tolerance in rice

Hong Zhang, Yanpei Liu, Feng Wen, Dongmei Yao, Lu Wang, Jin Guo, Lan Ni, Aying Zhang, Mingpu Tan, and Mingyi Jiang

SUPPLEMENTARY DATA

Supplementary Figure S1. Time courses of changes in the expression of *OsMPK4*, *OsMPK7* and *OsMPK14* in response to ABA treatment. The rice seedlings were treated with 100 μM ABA for various times as indicated. Relative expression levels of *OsMPK4*, *OsMPK7* and *OsMPK14* were analyzed by real-time quantitative RT-PCR. Values are means ± SE of three independent experiments. Means denoted by the same letter did not significantly differ at P <0.05 according to Duncan's multiple range test.

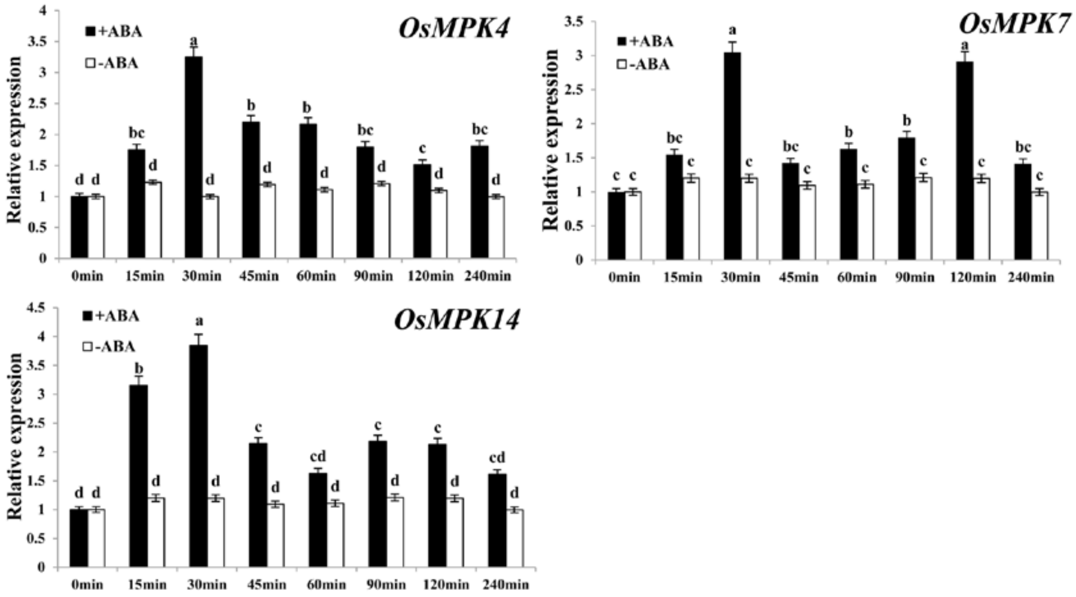

Supplement: Supplementary Data [file supp_eru313_jexbot121830_file001.pdf]
